# Salivary Biomarkers Associated with Psychological Alterations in Patients with Diabetes: A Systematic Review

**DOI:** 10.3390/medicina58081091

**Published:** 2022-08-12

**Authors:** Guillermo Bargues-Navarro, Vanessa Ibáñez-del Valle, Nisrin El Mlili, Omar Cauli

**Affiliations:** 1 Department of Nursing, Faculty of Nursing and Podiatry, University of Valencia, Avda Menéndez Pidal 19, 46010 Valencia, Spain; 2 Frailty and Cognitive Impairment Organized Group (FROG), University of Valencia, 46010 Valencia, Spain; 3 Chair of Active Ageing, University of Valencia, 4610 Valencia, Spain; 4 Higher Institute of Nursing Professions and Health Techniques (ISPITS), Tetouan 93000, Morocco; 5 Department of Biology and Health, Faculty of Sciences, University Abdelmalek Essâadi, Tetouan 93000, Morocco

**Keywords:** cortisol, melatonin, diabetes, saliva, sleep, depression, anxiety, stress

## Abstract

The care of individuals with diabetes needs a holistic perspective, taking into account both the physical disease and the mental health problems that may be associated. Different studies show a higher prevalence of depression or anxiety issues in diabetes patients than in the general population, which is why diabetes can be considered one of the chronic diseases in which psychological care is crucial to maintain quality of life. The objective of this review is to examine the published articles that relate the bidirectional associations between objective and subjective measures of anxiety, depressive symptomatology, stress, sleep quality, and salivary biomarkers in patients with diabetes. For this, a search was carried out in the electronic databases PubMed, Cochrane, and SCOPUS using the keywords “diabetes”, “saliva”, “sleep”, “anxiety”, “depression”, and “stress” for works published up until May 2022 and limited to the English and Spanish languages. The sample comprised 14 articles, 5 of which analysed the associations between depressive symptomatology and salivary biomarkers in people with diabetes. Among the salivary biomarkers most frequently used to evaluate psychological alterations in persons with diabetes are cortisol and melatonin. Thus, significant changes in the levels of these biomarkers were observed in most studies. Four out of five studies reported a statistically significant relationship between increased salivary cortisol in the evening/midnight or the cortisol awakening response and depressive symptoms. In contrast, lower cortisol levels upon waking in the morning were observed when there was no depression or anxiety. Regarding the association between salivary cortisol values and sleep quality in patients with diabetes, lower morning cortisol values related to prolonged nighttime sleep were common in the analysed studies. Low melatonin concentrations showed a negative correlation with sleep quality. As it is an easy-to-apply and non-invasive method, the measurement of salivary biomarkers can be very useful for predicting psychological alterations in patients with diabetes. Further scientific studies are required to determine the sensitivity of these biological substances acting as biomarkers for detecting sleep disorders and psychological alterations.

## 1. Introduction

Diabetes mellitus (DM) is a chronic, multi-causal disease that is currently increasing in incidence and prevalence in all age and sex ranges and regions of the world and is a growing concern for global public health [1,2]. It is estimated that by 2030, 552 million people worldwide will have DM [3]. Research on DM and its associated health problems is considered relevant because of its social and economic implications and the resulting challenges to health systems worldwide [1].

Among the health problems related to DM per se or its poor response or adherence to treatments are the many diseases that are caused by DM in other organs and tissues of the body such as cardiovascular disease [4], cancer [5], ocular diseases [6], kidney diseases [7], skin disorders [8], cognitive impairment [9,10], and diabetic neuropathy [11,12]. In relation to the effective management of DM, the main international organizations and health associations established the need to adopt a comprehensive health approach that takes into account not only the physical disease but also the mental health problems that are often associated with this pathology [13,14,15,16,17]. In the most common form of DM (type II DM (T2DM)), studies have shown a higher prevalence of depression- or anxiety-related problems compared to the general population (with an incidence of 40–50% for depression and anxiety disorders compared to less than 10% in both cases for the general population) [18]; therefore, DM can be considered one of the most psychologically and behaviorally demanding physical diseases due to its chronic condition.

In people with DM, it is not only important to know about glycemic and dietary control but also relevant to understand the psychological impact of the disease on the person, thus providing a comprehensive view of the disease. There are some relevant psychological conditions related to DM such as depression [19,20,21,22], major depression [23], major depressive disorder [24], stress [25], anxiety [21,22,26], and sleep disorders [27,28].

For the diagnosis, monitoring, prediction, prognosis, susceptibility, and risk of psychological disorders, the biomarkers for depression, major depression, anxiety, stress, and sleep problems can be used [29]. For example, dopamine for anxiety [26], depression [30], major depression [31], and sleep [32]; serotonin for anxiety [33], depression [34], and major depression [31]; cortisol and noradrenaline for stress [33]; and melatonin for sleep [32] are some of the biomarkers that can be used. 

In addition, compared to specimens obtained from the collection of blood, the analysis of saliva samples is useful for assessing markers associated with various diseases including endocrinological diseases, such as DM [34], obesity, inflammatory diseases, and psychiatric diseases, as well as for pharmacotherapy studies [35,36,37,38], and more recently for the reliable diagnosis of COVID-19 [39]. Many biomarkers identified in saliva samples have shown a significant correlation with biomarkers measured in blood-derived samples such as cortisol [40,41], testosterone, progesterone, and catecholamine [41]. Finally, saliva is easy to collect, can be self-collected, and offers the possibility of repeated measurements in a short period of time without being as invasive as venipuncture. Prior research has shown good correlations between salivary glucose (stimulated and unstimulated conditions) levels using different techniques and glucose levels in blood [42]. Glycated proteins such as HbA1C can be compared with salivary markers, such as serum cortisol levels, salivary cortisol, plasma, and prolactin levels [43]; other putative diabetic markers [44]; and the enzyme representing the first glycemic controlling enzyme in food digestion (i.e., salivary amylase) [45]. Moreover, fasting blood glucose and salivary glucose test scores have been correlated significantly in patients with DM [46,47] and there is, in turn, a positive correlation between fasting salivary glucose testing and HbA1c [46,48] and other salivary markers, for example, fructosamine-glycated protein showed a significant correlation with HbA1c and blood glucose [49]. Salivary amylase (the main enzyme involved in glucose release from saccharides) content is increased in diabetic patients compared to non-diabetic individuals [50]. The increase in salivary amylase concentration was generally observed in samples collected in fasting and non-fasting (measured 1 to 2 h after meal intake) conditions. The majority of the studies reported a threefold and higher increase in salivary glucose concentrations in individuals with DM, suggesting similar biochemical alterations were at the core of the increase in these two biomarkers of glycemic index in saliva. The increase in salivary glucose appears consistent and has been replicated in saliva samples collected both after fasting and non-fasting conditions. The aims of this systematic review were to analyse the relationship between the salivary biomarkers and psychological alterations in individuals with diabetes and to evaluate the differences based on the type of diabetes and other clinical factors such as the influence of the timing of the saliva sampling and the role of comorbidities or antidiabetic treatments. 

## 2. Methods

### 2.1. Literature Search 

In order to analyse the studies published on this topic, we conducted a literature review based on a systematic review design. Considering that the area of the salivary biomarkers and their relationship with psychological alterations in individuals with DM is relatively new, there are no published reviews on this topic to date. The methodology of the systematic review also provides the assessment of the quality of each selected study. A search for articles in electronic databases was carried out in May 2022, following the PRISMA guidelines. This search resulted in a review that included a total of 14 published studies. To carry out this search, we formulated the following research question: Is there any biomarker measured in saliva that can be associated with psychological alterations in individuals with DM? Based on clinical studies, we identified the most common psychological alterations reported in diabetic patients [19,20,21,22,23,24,25,26,27,28,50,51,52,53], e.g., depressive symptoms, anxiety, stress, and reduced sleep quality.

The search process was carried out in the following electronic databases: PubMed, SCOPUS, and Cochrane. We initially searched for potential primary studies in the databases using the following search string: “diabetes” AND “saliva” AND “depression” OR ‘‘sleep’’ OR “anxiety” OR “stress”. This search was designed following a keyword analysis of the available literature, which was obtained from several general searches of the resources listed above. 

### 2.2. Inclusion and Exclusion Criteria

The following inclusion criteria were applied: (1) full text in English or Spanish; (2) original research articles; and (3) identification of data regarding the concentration of any substance in saliva and its relationship with psychological alterations or symptoms. To determine which articles to include, we analysed their titles and abstracts. The full texts were retrieved for those that fulfilled the inclusion criteria. Finally, the reference lists for all the relevant articles were manually cross-referenced to identify any additional articles. The exclusion criteria were: abstracts or keywords not mentioning saliva/salivary and studies performed in species other than humans; and (3) articles written in languages that were not English or Spanish.

### 2.3. Evaluation of the Quality of the Methodology

The Agency for Healthcare Research and Quality (AHRQ) checklist was used to assess the quality of the included studies. We used the checklist for Cross-Sectional/Prevalence Studies since all the studies included in the review used these study designs [54]. 

## 3. Results

**Figure 1:**
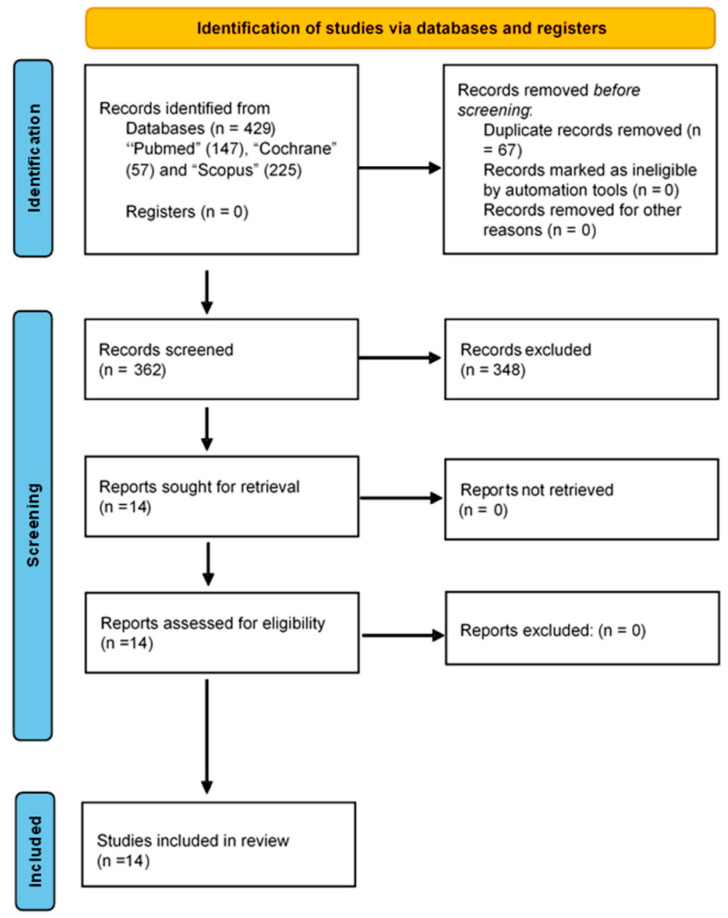
PRISMA flow chart.

As a result of the literature search process, 429 studies were identified. Excluding duplicate and unavailable results, 362 studies were obtained. Accordingly, 14 articles were selected and 348 articles were excluded. For all the primary articles included in the selection, the full draft of each paper was read in detail to decide whether to include or exclude the study. Finally, the primary articles included in the final selection were those that answered the research questions posed in this study. Figure 1 shows the PRISMA flowchart of the full review process. The many features of the selected studies are shown in Table 1. The participants in the studies selected were type I diabetes mellitus (T1DM) patients [55,56,57,58], T2DM patients [55,58,59,60,61,62,63,64,65], pregnant women with gestational diabetes mellitus (GDM) [66,67,68], and patients with diabetic retinopathy (DR) [55]. Figure 1 shows the sample characteristics and psychometric or clinical instruments used to analyse the psychological alterations and primary outcomes related to the salivary biomarkers and psychological alterations. 

### 3.1. Design of the Studies, Type of DM, and Salivary Biomarkers

In total, 14 articles were included who fulfilled the inclusion criteria. Of these, 11 were observational analyses of which 4 were cross-sectional analyses [57,59,64,67] and 7 were longitudinal [55,56,61,62,63,66,68]. In addition, three were experimental articles. Truninger et al., 2013 [58], was a study where participants completed a 2-hour driving training session exposed to mental stress.; Sanches et al., 2021 [60], was a study where participants partook in recreational training sessions for 2 months; and Adhikari et al., 2021 [65], was a study where participants self-administered supplementary light in the mornings for 30 min for 14 days. In total, information was collected and analysed from 522 people with T2DM, 376 people with T1DM, and 65 women with GMD. A total of eight articles studied people with T2DM [55,59,60,61,62,63,64,65], three studied people with T1DM [56,57,66], two studied women with GDM [67,68], and one article compared people with T2DM and people with T1DM [58].

Cortisol was analysed in 11 studies [55,56,57,58,59,60,61,62,63,66,67]; melatonin was analysed in 4 studies [55,64,65,68]; and the inactive metabolite of cortisone produced particularly in the kidneys was analysed in 1 study [66].

### 3.2. Biomarkers Analysed in the Selected Studies

The salivary biomarkers mostly analysed in the studies were cortisol and melatonin as crucial hormones involved in the chronic stress response and thus in depressive and anxiety symptoms and sleep, respectively. Cortisol as a circadian hormone was measured at different times of the day: in the morning in five studies [55,56,62,66,68], in the afternoon in one study [55], in the evening in four studies [55,56,58,62], and at night in three studies [55,62,67] (Table 2). Basal levels of salivary cortisol were reported in three studies [55,56,67] and they were similar to the concentrations in the control subjects, except for an increase in the diabetic patients in the evening [56]. No data were reported on the salivary concentration in the studies of Vedhara et al., 2010 [61]; Alvarez et al., 2013 [59]; Melin et al., 2014 [57]; Bawa et al., 2020 [63]; and Sanches et al., 2021 [60] since control groups were not included in the design of those studies. Salivary cortisol was studied in one article in people with T2DM [62], two articles in people with T1DM [66], one article in women with GDM [67], and one article in people with DM and diabetic retinopathy [55]. Salivary melatonin was studied in two articles in people with T2DM [55,64] and one article in women with GDM [68]. 

Melatonin was measured at different times of the day: in the early morning in three studies [55,64,68], the afternoon in two studies [55,68], the evening in two studies [55,68], and at night in two studies [55,68] (Table 3). Pregnant women with hypertensive or glucose metabolic disorders had smaller circadian variations in salivary melatonin secretion and their melatonin values were lower throughout the day than healthy pregnant women [68]. Regarding the salivary concentration of melatonin in T2DM patients and participants without T2DM, a significantly higher concentration was observed in the control group compared with the DM group [64]. Other biomarkers have been examined in other studies. Salivary cortisone (a metabolite of cortisol) was measured in one study in the morning [66]. In Table 3, no data about the manuscript of Adhikari et al., 2021 [65], was included because no control group was included in this study.

### 3.3. Associations between Salivary Biomarkers and Depression and Anxiety Symptoms

Five studies analysed the association between salivary biomarkers and depressive symptoms [57,59,60,61,62]. T2DM patients without depression had lower cortisol levels on waking in the morning than people without depression [62], and midnight salivary cortisol was associated with patients presenting with self-reported depression [57]. In patients with diabetic foot ulcers, depression was a statistically significant predictor of the likelihood of healing. Patients who were clinically depressed had fewer changes in ulcer sizes, and patients whose ulcers healed at 24 weeks from baseline were associated with lower evening cortisol and a higher awakening cortisol response at baseline [61]. After 12 weeks of recreational training (RET) in women with T2DM, salivary cortisol collected between 7:30 and 8:30 A.M. was lower as were reduced levels of T2DM-induced anxiety and depression [60]. In contrast, the 2013 study by Alvarez et al. [59] found no link between cortisol and depressive symptoms or depression in patients with T2DM. 

Three studies analysed the association between salivary biomarkers and anxiety symptoms in diabetic patients [60,61,66]. Anxiety did not predict changes in the ulcer of patients with diabetic foot ulcers [61], and in another study after adjustment for the analysis for the State-Trait Anxiety Inventory (STAI), there were no differences between the group of young patients with T1DM and the control group aged 6–12 years in salivary cortisol and cortisone, but the STAI score tended to be higher in the control group. No difference was observed between the T1DM and control groups in the Child Depression Inventory score [66].

### 3.4. Associations between Salivary Biomarkers and Sleep Quality and Stress

Four studies analysed the association between salivary biomarkers and sleep quality [56,64,65,68]. A lower cortisol awakening response (CAR) was associated with longer sleep [56]; nap duration was not associated with Dim Light Melatonin Onset (DLMO) [56]; and a lower melatonin concentration was negatively correlated with the PSQI score [64,68]. In contrast, these correlations were not observed in pregnant women with hypertensive or glucose disorder, either in the second or third trimester [68]. 

Three studies analysed the association between salivary biomarkers and stress [56,58,63]. Awakening cortisol levels and a blunted cortisol awakening response were significantly correlated with physical-related distress [63]; median salivary cortisol concentrations decreased during the control day and salivary cortisol concentrations increased on the stress driving test day [58]; and there was a significantly lower cortisol amplitude in the control subjects, and in contrast, salivary cortisol did not correlate with stress [56]. 

### 3.5. Differences between Type 1 and Type 2 DM 

Among all the studies analysed in this review, the one published by Ba-Ali et al. [55] was the only one that studied the circadian rhythm and sleep quality in diabetic patients, establishing three differentiated groups based on whether the participants were healthy or had been diagnosed with T1DM or T2DM. Patients with T1DM also had moderate diabetic retinopathy and patients with T2DM did not have DR.

Salivary cortisol and melatonin levels were measured, and activity and rest levels were assessed with actigraphy along with the subjective sleep quality scales, the (Pittsburgh Sleep Quality Index (PSQI) and Epworth Sleepiness Scale (ESS). The results on the daily variations of salivary melatonin showed a reduced amplitude. The mean level of melatonin in saliva had a maximum concentration at 04:00 A.M. both in healthy people and in people with T1DM and T2DM. The maximum concentration of melatonin at 04:00 A.M. was significantly lower in patients with both T1DM and T2DM than in healthy controls. The mean nocturnal melatonin concentration was also significantly reduced in both T1DM and T2DM compared to healthy controls. No significant differences were found in the mean nocturnal melatonin concentration or peak melatonin level between patients with T1DM and T2DM. There were also no significant differences in the mean nocturnal cortisol values or peak daytime cortisol levels between healthy controls and patients with T1DM and DR or patients with T2DM without DR.

With regard to the circadian rhythm assessed by actigraphy, this study showed more significant fragmentation of rest/activity intervals in patients with T1DM and diabetic retinopathy, indicating circadian misalignment. This intra-daily variability was not observed in patients with T2DM without diabetic retinopathy or in healthy participants. Neither the actigraphy sleep parameters nor the subjective PSQI or ESS scores significantly differed between the healthy controls and diabetic patients.

These data reveal a disturbance in the circadian rhythm in patients with DM, particularly those with T1DM and diabetic retinopathy. Melatonin plays an important role in the retina as an antioxidant molecule and a local modulator of the retinal circadian rhythm [69,70,71]. The reduced level of melatonin leads to a disturbed circadian rhythm and a higher level of free radicals, both of which precipitate retinal damage in diabetic patients. These findings suggest new research avenues aimed at studying oral melatonin use to improve synchronization and the circadian rhythm in diabetic patients.

### 3.6. Evaluation of the Quality of the Methodology

The AHRQ checklist is shown in Table 4. The first four items on the checklist were mostly analysed in the studies. The studies by Vedhara et al. [61] and Zahn et al. [62] have the highest methodology clarity (fulfilling seven out of eight items) based on the checklist items. Due to the type of design of the studies, only one of them clearly summarised patients’ response rates and the completeness of data collection (item 10). 

The items of the checklist are: 1.Define the source of information (survey, record review); 2. List the inclusion and exclusion criteria for exposed and unexposed subjects (cases and controls) or refer to previous publications; 3. Indicate the time taken to identify patients; 4. Indicate whether or not subjects were consecutive if not population-based; 5. Indicate whether the evaluators of the subjective components of the study were masked to the other aspects of the status of the participants; 6. Describe any assessments undertaken for quality assurance purposes (e.g., test/retest of primary outcome measurements); 7. Explain any patient exclusions from the analysis; 8. Describe how confounding was assessed and/or controlled; 9. If applicable, explain how missing data were handled in the analysis; 10. Summarise patient response rates and completeness of data collection; 11. Clarify what follow-up, if any, was expected and the percentage of patients for which incomplete data or follow-up was obtained.

## 4. Discussion

The analysis of the literature on the utility of salivary biomarkers for diagnosing or monitoring systemic diseases has begun to be of clinical use in several diseases including DM [72,73,74,75]. Besides the non-invasive nature of saliva sampling, biomarkers in saliva offer the possibility of sampling several times within the same day and as a consequence, saliva sampling could be easily carried out outside of hospitals or primary care centres. The overall concentration of cortisol in saliva can be modulated by stress and certain tasks but our review reveals that at the basal level, no significant differences were observed in the morning [55,56,67]. However, the concentration of salivary cortisol increased in individuals with type 1 DM under specific conditions, e.g., in women in the ovulation phase or individuals with preceding hypoglycaemia or with hyperglycaemia [56]. The amplitude of cortisol was reduced in the presence of perceived psychosocial stress but only in adult healthy controls and not in individuals with DM, suggesting the salivary cortisol concentration has limited use when assessing psychosocial stress in individuals with DM, mainly because of an overriding effect of the physiological metabolic stress of T1DM itself over psychosocial stress [56]. Under stress conditions, salivary cortisol did not change significantly compared to the changes in the control group [58,63]. Regarding the psychological alterations found in individuals with DM, depression is quite common [19,20,21,22,76], and depression has been associated with adverse outcomes in DM such as diabetic foot ulcer healing [61]. Even though some reports have found increased salivary cortisol associated with DM and depression, the association is absent or weak [55,57,59,62,66,67]. Cortisol in saliva (lower in the evening and higher at waking) has also been associated with longer foot ulcer healing [61]. However, salivary cortisol seems to be a good biological marker for the changes in psychological alterations after a programme of different non-conventional physical activities, music and breathing exercises, and games in older women with T2DM [60]. Salivary cortisol decreased as well as depressive and anxiety symptoms. Importantly, this effect was not due to a general metabolic improvement after the training programme since no effect was observed in circulating cholesterol, HbA1c, and proteinuria levels [60].

The role of salivary cortisol in other conditions associated with hyperglycaemia, such as chronic inflammatory state (e.g., in obesity, a common comorbidity in T2DM patients), warrants further studies. A recent meta-analysis concluded that diurnal cortisol slopes correlate with mental and physical health outcomes when inflammation and immune system alterations are present. Regarding salivary melatonin levels, the psychological parameter mostly studied is sleep quality. Although sleep quality indexes are related to the melatonin concentration in saliva, which makes them suitable for studying the neurological basis and the effects of intervention in sleep research, the studies did not find significant differences between DM and control individuals [55]

The peak salivary melatonin concentration in the early morning and the mean nocturnal melatonin concentration were significantly reduced in diabetic patients regardless of retinopathy stage [55] and in pregnant women [68]. The reduced nocturnal melatonin concentration and increased fragmentation of activity–rest intervals revealed circadian rhythm disturbances in diabetic patients with retinopathy [55,64]. Pregnant women with glucose metabolic disorder complications had smaller circadian variations in salivary melatonin secretion and reduced quality of sleep, and their values were lower throughout the day than healthy pregnant women [68]. As in the case of salivary cortisol, the salivary melatonin concentration seems to be a good marker for monitoring interventions to improve sleep quality. The effectiveness of supplemental light assessed by comparing subjective sleep questionnaire scores and salivary dim light melatonin onset before and after light exposure, as well as the use of a self-maintained sleep diary during light exposure, demonstrated that improvements in melatonin levels are in parallel with better sleep quality in individuals with T2DM [65].

Interestingly, the role of other DM alterations appears different and no changes in salivary melatonin concentration and rhythm were reported in those DM patients with nephropathy or neuropathy [64]. Although it did not include diabetic patients, a recent study analysed the relationship between glucose tolerance (whose alteration is a cornerstone of DM) and changes in melatonin in saliva [77]. At the time of awakening, participants with low glucose tolerance or “responders” had increased melatonin concentrations, but participants with high glucose tolerance or “non-responders” had increased melatonin concentrations in the middle of the night during the night of the slow-wave sleep suppression session. On the other hand, in relation to salivary cortisol, in the responder group, there was no effect observed during the SWS suppression session, but in the non-responders, there was a decrease in cortisol at 07:00 A.M., and a comparison of the two groups after the slow-wave sleep suppression session showed that the non-responders had higher salivary cortisol levels at 07:00 A.M. [77].

Emerging salivary biomarkers associated with DM risk during gestation have been recently described in the case of uric acid [78]. Salivary uric acid concentration has a robust circadian pattern throughout pregnancy with the highest concentrations at waking, a steep decline in the early morning, and decreasing levels throughout the day. It is associated with overweightness/obesity during pre-pregnancy (a well-known risk factor for gestational DM) and future studies should assess its changes in diabetic patients with or without pregnancy. Interestingly, lower salivary uric acid concentration has been associated with prior-night sleep duration and/or diurnal slopes [78]. There is a significant decrease in the total volume of saliva at rest in patients with type 1 [79] and type 2 [80] DM compared to individuals without DM. In addition, autoimmune diseases affecting salivary glands such as Sjögren’s syndrome [79,81] are more frequent in diabetic patients. These factors should be included when analysing salivary biomarkers in diabetic patients versus control groups since changes or the lack thereof in a given volume of saliva may not necessarily reflect that the overall production and volume of saliva secreted is lower in diabetic patients. Treatment of DM involves several types of drugs, and in the analysed studies this information was not available or where available, the effects of antidiabetic treatment on the concentration of salivary biomarkers were not assessed. This aspect clearly warrants further studies as these drugs can affect saliva production and/or cortisol/melatonin concentration.

These data suggest that future studies aimed at analysing the associations between salivary biomarkers and psychological alterations should take into account the modulating effects of long-term DM complications. 

## Figures and Tables

**Table 1:** Main characteristic of studies evaluating the relationship between salivary biomarkers and psychological alterations in DM.

Reference	Type of Study	Patients Characteristics Mean Age (Standard Deviation) (Sex/%/N)	Type of DM/Control (without DM)	Types of Psychological Alteration Analysed	Instrument Used to Measure Psychological Alterations	Type of Salivary Biomarkers	Main Results
**Vedhara et al., 2010** [61]	Observational	60.7 (11.0) years old Male: 73.1 (68) Female: 26.9 (25)	T2DM: 93	Depression	Hospital Anxiety and Depression Scale (HADS) questionnaire	Cortisol. Collection of four saliva samples over a single day: at waking, 30 min later, between 11 A.M. and 13 P.M. (before lunch) and between 20 and 21 P.M. (at least 2h after the evening meal)	Depression and anxiety symptom scores were a statistically significant predictor of the likelihood of diabetic foot ulcer healing. Patients in whom ulcers healed at 24 weeks were associated with lower evening cortisol and higher cortisol response at awakening at baseline.
**Truninger et al., 2013** [58]	Experimental	55 (12) years old Male: 79.5 (31) Female: 20.5 (8)	T2DM: 21 T1DM: 18	Stress	Subjective stress perception was determined with a Visual Analogue Scale (VAS)	Cortisol. Collection of six saliva samples over a single day: 30 min before and 30, 75, 120, 180, and 240 min after the start of the driving training, during the evening	Insulin-treated people with DM were subjected to a 2 h stress period to observe the effects of stress on glucose control. Salivary cortisol levels increased significantly on the day of the stress test and decreased on the control day. However, there were no significant changes in glucose concentrations when comparing the control day with the sustained stress day.
**Alvarez et al., 2013** [59]	Observational	62 (7) years old Male: 49.2 (30) Female: 50.8 (31)	T2DM: 61	Major Depressive Disorder	MDD according to Diagnostic and Statistical Manual Disorder criteria (MSD-IV), Hamilton Rating Scale for Depression (HA.M.-D17), Mini International Neuropsychiatric Interview (M.I.N.I), and Beck Depression Inventory (BDI)	Cortisol. One saliva sample was collected at 23.00 P.M.	Higher late-evening salivary cortisol in individuals with depression but the difference was not significant. Saliva cortisol levels at 23:00 h were higher in patients with depression and DM.
**Melin et al., 2014** [57]	Observational	41.3 years old Male: 54.1 (106) Female: 45.9 (90)	T1DM: 196	Depression	Self-reported depression assessed by Hospital Anxiety and Depression Subscale (HADS) questionnaire, medical records of depression	Cortisol. One sample was collected at midnight.	Self-reported depression was associated with midnight salivary cortisol (MSC).
**Zahn et al., 2015** [62]	Observational	T2DM and Major Depression [MD] group: 53.8 (3.6) years old T2DM group: 57.9 (8.9) years old MD group: 48.8 (3.7) years old Control group: 53.7 (4.8) years old Female: 100 (84)	T2DM: 22 Control: 24	Major Depression	Structured clinical interview for the diagnosis of psychiatric disorders according to DSM-IV-TR criteria. Severity of depression: quick inventory of depressive symptomatology	Cortisol. Collection of six saliva samples: immediately after awakening, 30 min, 4 h, 8 h, 12 h and 16 h later for the diurnal decrease in salivary cortisol concentrations (slope) analysis	Depressed patients had marginally lower cortisol than healthy controls, whereas comorbid patients and MD patients had similar cortisol awakening response levels. No effect on major depression, T2DM, or interaction with cortisol levels.
**Horsch et al., 2016** [67]	Observational	Gestational diabetes mellitus [GDM] group: 32.7 (5.2) years old Control group: 29.7 (5.5) years old Female: 100 (203)	GDM: 39 Control: 164	Perceived stress, subjective experience of stress, anxiety, depression, sleep	Perceived Stress Scale (PSS)	Cortisol. One sample of fasting morning saliva between 7:30–9:30 A.M. at least 90 min after awakening and the other at around 10 P.M. per person, for cortisol analysis	There were no significant differences between women with or without GDM with regard to morning or evening salivary cortisol.
**Shimada et al., 2016** [68]	Observational	Pregnant women with pregnancy complications group (hypertensive or glucose metabolic disorder): 35.1 (2.4) Pregnant women without pregnancy complications group: 33.3 (3.4) Female: 100 (98)	Control: 40 Pregnant women with hypertensive and glucose metabolic disorder: 7 Pregnant women with glucose metabolic disorder: 19 Pregnant women with hypertensive disorder: 31	Sleep	Pittsburgh Sleep Quality Index (PSQI) and quantifying sleep logs using a sleep questionnaire where participants recorded their sleep times every 30 min for 1 week.	Melatonin. Daily collection of four samples for 3 days: before breakfast, lunch, dinner and before going to sleep	Pittsburgh Sleep Quality Index (PSQI) sleep efficiency had a negative correlation with night and amplitude melatonin levels in third-trimester healthy pregnant women.
**Ba-Ali et al., 2019** [55]	Observational	Control group: 60 (10) DM without diabetic retinopathy group: 63.1 [3.8] DM and moderate DR group: 61.6 (1.9) Male: 52 (39) Female: 48 (36)	Diabetic patients with moderate DR: 25 Diabetic patients without DR: 29 Control: 21	Sleep	Pittsburgh Sleep Quality Index (PSQI) and Epworth Sleep Scale (ESS). Objective methods: actigraphy to assess circadian rhythm and quantify exposure to light.	Melatonin and cortisol. Seven saliva samples were collected every 4 h for 24 h.	No correlation between melatonin or cortisol levels with Pittsburgh Sleep Quality Index (PSQI) and Epworth Sleep Scale (ESS) scores
**Kalere et al., 2019** [64]	Observational	Age range: 26 to 86 years old Male: 18 Female: 20	T2DM: 26 Control: 12	Sleep	Pittsburgh Sleep Quality Index (PSQI)	Melatonin. One sample of unstimulated saliva was collected after awakening (6–6:30 A.M.).	Trend in negative correlation of Pittsburgh Sleep Quality Index (PSQI) score with melatonin concentration but it was not significant
**Bawa et al., 2020** [63]	Observational	63.6 (6.9) years old Male: 63.4 (85) Female: 36.6 (49)	T2DM: 134	Depressive symptoms Distress	DM Distress Scale	Cortisol. Collection of five saliva samples over a single day: upon waking, thirty minutes after waking, at 10:00–10:30, 16:00–16:30, and 20:00–20:30	There was no significant association between DM-related distress with evening cortisol and cortisol awakening response
**Kristiansen et al., 2020** [56]	Observational	Children/adolescent control group: 14 (2.4) years old Children/adolescent T1DM group: 13.7 (2.8) years old Adult control group: 36.7 (1.1) years old Adult T1DM group: 44.3 (1.1) years old	T1DM: 113 Control: 54	Sleep	Self-reported times for going to bed and awakening, Self-reported psychosocial stress (yes/no and duration)	Cortisol. Three saliva samples were collected on two days: the first day in the morning immediately after awakening and the evening before going to sleep; the second day in the morning immediately after awakening.	More sleep time reduces cortisol awakening response, and the presence of perceived psychosocial stress reduces the amplitude of salivary cortisol in healthy adults. No cortisol markers correlated with perceived psychological stress in diabetics groups.
**Adhikari et al., 2021** [65]	Experimental	64.5 (6.9) years old Male: 70 (7) Female: 30 (3)	T2DM: 10	Sleep	Major depressive disorders using the Center for Epidemiologic Studies Depression Scale (CES-D), Pittsburgh Sleep Quality Index (PSQI), Epworth Sleep Scale (ESS), sleep diary, chronotype assessed with the morning–evening questionnaire, actigraphy to record light exposure.	Melatonin. Seven saliva samples for melatonin analyses and estimate Dim Light Melatonin Onset (DLMO) under dim room light, during the night.	Salivary dim light melatonin onset (DLMO) with light exposure strengthens sleep and decreases sleep fragmentation.
**Brossaud et al., 2021** [66]	Observational	Control group: 9.0 (1.7) years old T1DM group: 9.3 (1.4) years old Male: 52 (39) Female: 48 (36)	T1DM: 49 Control: 26	Anxiety Depression	State-Trait Anxiety Inventory (STAI), Child Depression Inventory (CDI)	Cortisol and corticosterone. Two saliva samples were taken at awakening and 30 min after awakening for cortisol analysis over 5 consecutive days.	There was no difference between the T1DM patient group and control in salivary cortisol and salivary corticosterone levels after adjusting the analysis for the State-Trait Anxiety Inventory (STAI). The STAI score tended to be higher in the control group. No difference was observed between the T1DM and control groups in CDI score.
**Sanches et al., 2021** [60]	Experimental	71 (2.9) years old Female: 100 (101)	T2DM: 101	Anxiety Depression	Hospital Anxiety and Depression Scale (HADS) questionnaire	Cortisol. Two saliva samples were collected between 7:30 A.M. and 8:30 A.M., 1 before recreational training protocol (RET) initiation and 1 after post-RET training	Lower salivary cortisol, and reduced T2DM-induced anxiety and depression levels after 12 weeks of RET.

T1DM: type 1 diabetes mellitus; T2DM: type 2 diabetes mellitus; GDM: gestational diabetes mellitus.

**Table 2:** Changes in salivary cortisol concentration in diabetic patients compared to control group.

Reference	Morning	Afternoon	Evening	Night
Zahn et al., 2015 [62]	Cortisol awakening response, Mean (SD): T2DM and Depression 8.6 (10.1) T2DM: 4.79 (8.1) Depression: 7.4 (9.7) Control: 13.6 (10.4)	Cortisol slope, Mean (SD) T2DM and Depression: 0.59 (0.39) T2DM: 0.69 (0.37) Depression 0.79 (0.36) Control 0.70 (0.34)		
Horsch et al., 2016 [67]	With GDM (Mean, SD): 29.82 (15.76) nmol/L without GDM: 28.84 (12.80) nmol/L		With GDM Mean (SD) 0.70 (0.21) nmol/L without GDM 0.72 0.22 nmol/L	
Brossaud et al., 2021 [66]	Control: 3.61 [3.02;4.37] (nmol/l) T1DM: 3.59 [2.81;4.21]			
Ba-Ali et al., 2019 [55]	Peak diurnal (08:00 A.M.) Control: 22.8 ± 10.9 mg/dL No T2DM retinopathy: 24.4 ± 11.6 mg/dL With T2DM retinopathy: 18.9 ± 8.9 mg/dL			Cortisol Average nocturnal Control: 12.0 ± 7.7 mg/dL No T2DM retinopathy: 11.3 ± 3.9 mg/dL With diabetic retinopathy: 11.6 ± 3.7 mg/dL
Kristiansen et al., 2020 [56] sample: children	Mean (SD): Control 7.9 (3.1); T1DM 8.6 (3.7) mmol/L Median (range): Control 7.7 (1.5–16.5); T1DM 7.9 (1.7–21.0) mmol/L		Mean (SD): Control 1.0 (1.1); T1DM 1.2 (1.1) mmol/L Median (range): Control 0.7 (0.2–6.8); T1DM 1.0 (0.2–7.4) mmol/L	
Kristiansen et al., 2020 [56] sample: adults	Mean (SD): Control 10.0 (5.0); T1DM 8.2 (4.2) Median (range): Control 8.9 (2.6–20.9); T1DM 7.4 (2.2–22.1) mmol/L		Mean (SD): Control 1.0 (0.5); T1DM 1.4 (1.1) Median (range): Control 1.0 (0.3–2.1); T1DM1.2 (0.3–5.8) mmol/L	

T1DM: type 1 diabetes mellitus; T2DM: type 2 diabetes mellitus; GDM: gestational diabetes mellitus.

**Table 3:** Changes in salivary melatonin concentration in diabetic patients compared to control group.

Reference	Morning	Night
Ba-Ali et al., 2019 [55]	Peak diurnal melatonin pg/mL (04:00) Control: 11.4 ± 10.7 No T2DM retinopathy 4.8 ± 5.3 *p* = 0.01 With T2DM retinopathy 2.9 ± 2.6 *p* < 0.001	Melatonin Average Nocturnal pg/mL: Control: 5.5 ± 4.0 No T2DM retinopathy: 3.2 ± 3.8 *p* = 0.01 With T2DM retinopathy 1.7 ± 1.4 *p* < 0.001
Shimada et al., 2016 [68]	Basal values were estimated from graphics since they were not reported in the results section. In third trimester healthy pregnant women had higher salivary melatonin levels (4 pg/mL at 8:00 A.M., 34 pg/mL at 13:00 P.M., 16 pg/mL at 19:30 P.M., 13 pg/mL at 23 P.M.) and third trimester pregnant women with complications (14 pg/mL at 8:00 A.M, 5 pg/mL at 13:00 P.M., 3 pg/mL at 19:30 P.M., 19 pg/mL at 23:00 P.M.)	
Kalere et al., 2019 [64]	Control group: 17.8 (8.2; 25.5) pg/mL vs. T2DM 6.1 (0.78; 12.2) pg/mL	

T1DM: type 1 diabetes mellitus; T2DM: type 2 diabetes mellitus; GDM: gestational diabetes mellitus.

**Table 4:** The Agency for Healthcare Research and Quality (AHRQ) checklist was used to assess the quality of the included studies.

Article\Items	1	2	3	4	5	6	7	8	9	10	11
Vedhara et al., 2010 [61]	Y	N	Y	Y	U	N	Y	U	Y	Y	Y
Truninger et al., 2013 [58]	Y	Y	N	Y	Y	Y	Y	U	U	U	U
Alvarez et al., 2013 [59]	Y	N	Y	N	U	Y	U	U	U	U	Y
Melin et al., 2014 [57]	Y	Y	Y	Y	U	N	Y	N	U	U	Y
Zahn et al., 2015 [62]	Y	Y	Y	Y	U	N	Y	Y	Y	U	U
Horsch et al., 2016 [67]	Y	N	Y	Y	U	Y	Y	U	Y	U	U
Shimada et al., 2016 [68]	Y	N	U	Y	U	Y	N	Y	U	U	U
Ba-Ali et al., 2019 [55]	Y	Y	N	Y	U	Y	N	N	U	U	U
Kalere et al., 2019 [64]	Y	N	N	N	U	N	N	U	U	U	U
Bawa et al., 2020 [63]	Y	N	Y	Y	U	N	Y	Y	Y	U	U
Kristiansen et al., 2020 [56]	Y	Y	Y	Y	U	Y	Y	U	U	U	U
Adhikari et al., 2021 [65]	Y	Y	Y	Y	U	N	Y	U	U	U	U
Brossaud et al., 2021 [66]	Y	Y	N	Y	U	N	N	U	U	U	U
Sanches et al., 2021 [60]	Y	Y	N	Y	U	N	U	U	U	U	Y

Y: yes; N: No; U: Unclear.
